# Medical Opinion and Movement

**Published:** 1909-12-11

**Authors:** 


### Medical Opinion and Movement.

At a meeting of the Société Médicale des Hôpitaux a case of Aortic Dilatation was shown in which the tracheal sign was the inverse of that usually described. The classic sign consists in a rhythmic depression of the laryngo-tracheal tube, drawn down by the synchronous pulsations of the artery. The presence of this sign is diagnostic of an aneurysm of the postero-inferior end of the transverse portion of the aorta. The left bronchus situated under the concavity of the aortic arch is depressed at each pulsation of the aneurysm and transmits the descending oscillations to the trachea and larynx. In the present case, according to Dr. Hirtz, the inverse sign would be due to the presence of an aneurysmal sac on the convexity of the aorta instead of on the concavity. The sac pushes the trachea from below upwards and transmits to it ascending oscillations instead of descending. The presence of the aneurysm was confirmed by radiography.

An interesting case of a Supernumerary Ureter, opening at the vulva, is placed on record by Dr. C. D. Josephson in the *Zentralblatt für Gynäkologie*. The patient was a young girl aged 18, who appeared to be suffering from incontinence of urine. Careful examination, however, revealed below and to the left of the urinary meatus a small aperture from which flowed drop by drop a little clear urine. Cystoscopic examination and catheterisation of the ureters showed nothing abnormal. Injection of methylene blue into the renal pelves did not lead to a coloration of the urine from the fistula, but when injected subcutaneously there was coloration of the urine both from the bladder and from the fistula. Abdominal palpation disclosed nothing further, and on the assumption of a supernumerary ureter from the left kidney, this was exposed by a lumbar incision after passing a catheter up the left ureter. The kidney was found to be longer than normal, the upper end being separated from the rest by a transverse furrow. Extending from this upper part was found a supernumerary ureter, 1 centimetre in diameter. This ureter was ligatured, resected, and the stump cauterised. The upper part of the kidney was excised along the furrow. The tissue here was found to be fibrous and its division caused no hæmorrhage. A small cystic cavity was disclosed surrounding the upper end of the kidney, rupture of which caused the flow of a little purulent fluid. A gauze-drainage was placed in the wound. At the end of three weeks there was only a small fistula in the lumbar region, the pseudo-incontinence had, of course, completely disappeared.

At a meeting of the *Académie de Médecine*, Professor Metchnikoff gave an account of his researches on the Pathology of Infantile Diarrhœa. Animals suckled at the breast develop a diarrhœa often fatal when made to ingest a small quantity of the fæces from infants suffering from gastroenteritis. His experiments were made with rabbits and monkeys, and they clearly demonstrated the infective nature of the disease. The next step was to discover the causal agent. Rabbits and monkeys were then fed with pure cultures of the Bacillus Proteus isolated from the fæces of infants affected with the disease, and also from one of the monkeys in the previous experiments. All the animals proved sensitive to the infection, but instead of developing the usual diarrhœa they showed symptoms characteristic of dry cholera, which, however, proved fatal. The bacillus was recovered again in the intestinal contents, and also sometimes in the blood contained in the heart. The author concludes from these experiments that the Bacillus Proteus is a causal agent of infantile diarrhœa. He points out that this microbe is rarely found in cow's milk, and further that infants fed at the breast not infrequently develop the disease. The infection must be conveyed therefore in some other way, probably through individuals nursing the infants. He suggests that in the summer the food becomes contaminated by the microbe, being carried hither from animal excreta by the flies. In this way adults absorb large quantities of these microbes with their food, and eventually the infants become contaminated. The author advocates, therefore, the careful cleansing of the breasts and of the hands and other precautions against contamination of food with the Bacillus Proteus.

The number and variety of purposes for which the introduction of Medicaments by the Electric Current may be utilised continue to increase, and there seems little doubt that in ionotherapy we have a most useful means of treatment for all manner of affections of the skin. In *La Semaine Médicale* appears an article by Dr. Ed. H. Blanc, of Paris, on the cleansing and disinfection of the sebaceous glands by ionisation. In cases of infection of the glands by the *Demodex folliculorum*, or by the seborrhœic micro-bacillus of Sabouraud, or by other saprophytic and pathogenic microbes, the difficulty of penetrating the glands and disinfecting them by the application of powders, ointments, and lotions is only too familiar to dermatologists and general practitioners, and cases of acne, seborrhœic dermatitis, and the like are among the most intractable to ordinary methods of treatment. Ionisation, of course, involves the expenditure of some considerable time and attention, but this would hardly prove a bar to its adoption if it be found to be really efficacious. The medicaments employed by Dr. Blanc are sodium salicylate, sodium iodide, and sodium hyposulphite in 1 per cent. solution. A lint pad is soaked in the solution and attached to the negative electrode of the battery, and this is applied to the affected part. The positive pole, consisting of another pad soaked in normal saline, is applied to some indifferent part of the body. The strength of current should be about 10 milliampères, and the duration of the *séance* 10 to 12 minutes. Eight or ten applications at the most are requisite to effect a cure. In cases in which the *Demodex folliculorum* is present the hyposulphite of soda is found to be especially efficacious. The iodides have a powerful antiseptic action, but they are liable to cause some irritation of the skin and also a temporary discoloration at the glandular orifices. For the generality of cases, therefore, the author gives the preference to sodium salicylate. Apart from the chemical action, the electric current also exercises a stimulating effect upon the muscular fibres of the skin, increasing their tone and so preventing the stagnation of sebaceous matter in the glands.

The Cremasteric Reflex has been studied with much energy by Mr. E. M. Corner and his clinical assistants, and the results of their researches are published in the *British Journal of Children's Diseases*. Considering the length of time that this reflex has been known, it seems remarkable that so many things connected with it should have been hitherto unknown, or at least unpublished. According to this author, the reflex usually first appears during the second year of life: it is never present at birth, but may be found as early as two months of age, or not until eight years in certain cases of defective mental development. When once it appears it soon becomes so brisk that the testicle may appear to be imperfectly descended. It is not confined to boys, for a corresponding inguinal reflex is common in girls; in the latter it is especially brisk about the ages of seven to twelve, after which it recedes, and is often completely lost. In boys, on the other hand, the reflex is frequently sluggish and feeble between these age limits, re-appearing actively again when puberty is reached: but the greatest vigour of the reflex is about six and seven years of age. Later in life it is gradually lost. The cremasteric reflex may be obtained in a variety of ways: thus stimulation, not only of the inner side of the thigh, but of the abdomen, perinæum, buttocks, prepuce, and even of the outer side of the thigh may produce it. Further, the response itself varies: not only may the testicle of the side stimulated be drawn up, but a similar result may affect the other side, though usually in a lesser degree; and yet again the response may occur only on the side opposite to that stimulated. At other times there is seen a general abdominal muscular contraction, so that the cremasteric becomes part and parcel of the abdominal reflex.

The practical applications of these physiological observations are numerous. Thus the habitual stimulation of the clothes may, in those of abnormally brisk cremasteric reflex, account for undescended testicles; and as a fact it sometimes happens that testicles will descend into the scrotum of a child lying naked in bed which had been supposed to be hopelessly retained. The same result may, of course, occur when an anæsthetic is administered for operation on such cases. The reflex is most brisk in healthy children, and may therefore be of use in detecting malingering. In the early stages of rickets, before bone changes are seen, it is weak or even abolished. It is temporarily abolished after operations on the inguinal region, and for a longer period in a suppurating than in a clean case. When the inguinal canal is sewn up in a radical cure of hernia, Mr. Corner believes that if the reflex returns in 10 days or a fortnight some of the stitches in the canal have not held; that if it is lost for more than four weeks (the wound healing by first intention), then the spermatic cord has been injured or there is deep suppuration; and that if the reflex returns in two or three weeks the operation has been a success. After removal of a varicocele the reflex is absent for a much longer time than after radical cure. In spastic conditions the cremasteric reflex is sometimes unaffected, though at others the testicles are retracted by tonic contractions of the muscle. For the applications of the results to the difficult questions connected with cryptorchism the original paper should be consulted.

Professor Leonard Rogers contributes to the *Therapeutic Gazette* a summary of his researches and experience upon the treatment of Cholera by Hypertonic Transfusion. He first established the fact that in this disease there is a tremendous loss of blood serum without much corresponding destruction of the corpuscles. The specific gravity of the blood rises from 1056 to 1070, and even more; the corpuscular volume rises from 45 per cent. to 60, 70, and, rarely, 80 per cent. Investigation also shows that in the severest fatal cases the average loss of serum equals 64 per cent. of the total; in the severe cases which recover after transfusion it averages 52 per cent.; and in milder cases not requiring transfusion it is about 35 per cent. A still surer guide is the blood-pressure, nominally about 100 to 110 mm. of mercury in the Hindu; if this falls below 70 mm. it indicates the necessity for intravenous transfusion. The cause of the concentration of the blood by such large doses of serum is, of course, the frequent and copious liquid stools and the vomiting which are characteristic of the disease. As a control over the quantity of fluid to be injected the blood-pressure and the specific gravity of the blood are both of use; a pressure of 100 mm. is to be aimed at, though it is not always possible to secure it. The pulse is a vary fallacious guide if estimated by the finger alone, for it often improves greatly after one or two pints of transfusion, whereas four pints is about the least that affects the blood-pressure, and the patient's chance of recovery, satisfactorily.

For not very severe cases rectal saline injections often do good, but the patient must be very carefully watched; continuous proctoclysis has lately been tried with good results. Subcutaneous transfusion Professor Rogers has given up, though he admits it is useful in certain cases, in favour of the more efficient intravenous method. This latter plan has been extensively tried for years, with results on the whole disappointing; and the author's experience was also unsatisfactory until he began to use hypertonic solutions. He points out that the percentage of salts in the blood is so much raised that normal saline tends rather to leave the vessels than to stay in them, and so favours diarrhœa. He uses a solution of 120 grains of sodium chloride with

3 grains of calcium chloride to the pint, practically double the usual saline strength, with the calcium salt added to increase the coagulability of the blood. After the first three or four pints the calcium chloride is left out. In 1908 and 1909 278 cases have been thus treated, with a mortality of 33.8 per cent., compared with 61.2 per cent. for 417 cases in 1902-1907. Moreover, but 14 per cent. of the 278 died in collapse, the reduction of mortality being much greater here than in the other stages of the disease. Professor Rogers is very sanguine that he will shortly be able to reduce his death-rate a good deal more.

---

PRIMARY Ovarian Pregnancy is now generally admitted to be a fact, though one of very extreme rarity. It has remained for a St. Louis physician, Dr. Kirchner, to record such a case in which the ectopic pregnancy has gone to term and mother and child have both been saved. The case is too long to rehearse in detail, but briefly the author delivered by laparotomy a full-term male mulatto child, of 6¾ lbs. weight, which is, at the age of 10 months, still thriving. With the exception of a very slight asymmetry of the head, the infant is a normal one. The points upon which the diagnosis rest are as follows: A gestation sac, in which were the fœtus and placenta, was found at operation. It was at first taken for an ovarian cyst, and its pedicle, with its blood supply, arose from the region where the right ovary should have been. The uterus was free, slightly enlarged, and prolapsed into the vagina. The left ovary and tube were normal. No right ovary was found, but the right tube was adherent externally to the gestation sac, as was also the appendix; apart from this the tube was normal. The placenta was entirely within the gestation sac, it had no relation with either tube nor with any part of the peritoneum. Nothing indicative of tubo-ovarian or abdominal pregnancy could be found. The physical characters of the sac are those of an ovarian cyst, and microscopically there is evidence of ovarian structure and origin. This combination seems sufficient to convince the most sceptical that the pregnancy really was ovarian.

---

AMONG the advances towards perfection which have been so frequently made in the last decade in the diagnosis of ureteric and renal lesions, the Early Detection of Tuberculosis of the Urinary Tract is one of the most notable. How far the examination of materials obtained by catheterisation of the ureters is to be regarded as absolutely diagnostic is not yet quite settled; but a remarkable case reported in the *Annals of Surgery* shows to what a high efficiency modern bacteriological analysis has reached. Briefly, the patient was a man who was under treatment for frequent and painful micturition attributed to the results of long antecedent gonorrhœa. Examination of the urine showed leucocytes and tubercle bacilli in an acid urine, and cystoscopy with ureteric catheterisation determined that these came from the left kidney. The latter was accordingly exposed by operation, but appeared normal. While separating some adhesions, the surgeon tore the pedicle so badly that the bleeding could be controlled only by clamps: when this was done he split the kidney, but could find no macroscopic evidence of disease. Failing to secure the bleeding points by ligature, he was constrained to remove what he thought was a normal kidney. Afterwards a small caseous focus was found, and the scrapings from this contained tubercle bacilli. The significant lesson is that if, in any patient with urinary symptoms and signs, tubercle bacilli can be proved to exist in the urine obtained by ureteric catheterisation, immediate nephrectomy is indicated without any division of the kidney. This particular patient developed a local infection of the wound, which resulted in a long-persisting sinus; and he also had to undergo castration six months later for tuberculous epididymitis: from these complications he has now recovered.

---

IT has for a long time been held, on the strength apparently of an American author, that deaf mutes rarely, if ever, learn to swim, and that it is very difficult, if not impossible, to teach them to do so. The matter was referred to at the recent French Congress of Otology in a paper by Moore and Cauzard on recent improvements in our methods of diagnosing labyrinthine disease. Mr. Dan Mackenzie, however, in the *Journal of Laryngology*, denies this supposed disability, though it would seem that deaf mutes experience exceptional difficulty in learning to swim under water. The physiological, or pathological, explanation of such cases is, no doubt, to be sought in lesions of the vestibular apparatus or the semicircular canals. Thomas has found that dogs cannot maintain their equilibrium in water if their vestibular nerves have been cut, though they do so easily when the cerebellum is destroyed but the nerves are left intact. It also appears, from an account which Mackenzie gives of a letter received from the headmaster of an institution for the deaf and dumb, that, in point of fact, deaf mutes swim easily and well; more so, indeed, than do normal children. The conflict of evidence may well be supposed to have arisen from generalisations on insufficient experience: that is to say, the idea of inability probably arose from experience gained from a few children in whom the vestibular end-organs were not functional. On the other hand, there would seem to be some mechanism by which the function of equilibration can be performed by the education and compensatory development of some other structure, not the vestibule: for some deaf mutes are able in course of time to acquire powers of walking in the dark which at first they do not possess, and this is most probably by training their tactile and muscular senses to replace the missing vestibular impressions. But in the majority of deaf mutes there is no question of any destruction of the vestibular end-organs or nerves, and in these it is certainly hard to understand how there can be any difficulty in swimming, either on the water or beneath it. The point is one which wanted ventilating, and Mr. Mackenzie's account seems to have settled it in the way one would naturally expect.

